# COMMENTARY ON: “A Calculus “Toy” in the Bladder. A Case Report of Rare Entity and Comprehensive Review of the Literature”

**DOI:** 10.15388/Amed.2022.29.1.15

**Published:** 2022-06-21

**Authors:** Jihad El Anzaoui

**Affiliations:** Urology department, Military Hospital My Ismail, Morocco; Email: jihad.elanzaoui@usmba.ac.ma

**Keywords:** Jackstone, bladder stone, lithogenesis, calcium oxalate

The article entitled: Jackstone: A Calculus “Toy” in the Bladder. A Case Report of Rare Entity and Comprehensive Review of the Literature [1], is as far as we know the first medical review concerning Jackstone (JS) in the literature.

Even if JS are rarely encountered in daily practice, their geometrical form is so bizarre and sometimes amazingly symmetric that their existence raises some questions about their genesis and clinic-therapeutic features.

The authors of the review were specifically interested in bladder JS, albeit this type of stone can be also renal.

We thought that at least five cases of renal jackstone were reported in the literature. Undoubtedly, renal location deserves consideration and study.

As we have learned from the review, the bladder JS behaves like any other bladder stone without a specific clinical or therapeutic aspect.

This review did not report the outcomes of stone composition analysis. We believe that the main reason is that it was not specified by the majority of reported cases.

While the pathophysiological features of JS are the most unknown and attractive, unfortunately, it seems that they are the less studied in the literature.

The geometrical shape emanates questions about physicochemical composition and growth model more than any other consideration.

A most recent study [2], cited by Simeonidis’s review, represents in our opinion, a turning point in the history of JS study.

V.H. Canela et al. submitted 98 JS to three investigations: micro-computed tomography, infrared spectroscopy, and immunohistochemistry analysis.

The study revealed three principal findings:

1. Structural findings: all of JS consisted of a central piece called “body” and one or numerous lateral extensions called “arms” of the stone.
  Whether the body or the arms are composed of two parts: a proteic radiolucent nucleus and a mineral shell.
  While the nucleus is mainly composed of Tamm–Horsfall protein, the mineral shell incorporates concentric external layers of monohydrate form of calcium oxalate (COM).
  The body shell contains a unique and thin layer of apatite that does not exist in the arm.
  Thus, the shell of the arms does not contain apatite but only many layers of COM as extensions of body layers.
2. Physio-dynamic findings:
  The shell of the arm is just a tapered continuation of the body shell. The disposition of the shell layers indicates that the arm grows faster than the body of the stone.
3. Etiological findings: the authors try to explain the birth and the growth of the arms.
  They hypothesize that arms develop in areas subject to physical abrasion of the mineral coating. That permits aggregation of proteic constituents to the nucleus tip and fixation of the mineral coating progressively as far as the arm grows.

The consistency of this study is highlighted by analyzing a great collection of JS that exceeds the sum of all reported cases in the literature.

Although the origin of these stones was not identified (vesical or renal), and the study did not certainly reveal all JS mysteries, it has considerably enriched our knowledge about the lithogenesis of JS.
